# Meta-Analysis of Data from Radiometric Partial Discharge Localization Systems

**DOI:** 10.3390/s26144353

**Published:** 2026-07-09

**Authors:** Allan D. C. Silva, Raimundo C. S. Freire, Luiz A. M. M. Nobrega, Itaiara F. Carvalho, Luiz F. B. Alves, Victor F. B. Mendonça, Edmar C. Gurjão

**Affiliations:** Department of Electrical Engineering, Universidade Federal de Campina Grande, Aprigio Veloso 882, Campina Grande 58429-900, Brazil

**Keywords:** high-voltage equipment, localization estimation, received signal strength

## Abstract

Partial discharge localization is a crucial step in monitoring high-voltage equipment. Over time, due to data acquisition limitations, signal strength has become a key driver of methods of localization. In the literature, the variety of testing conditions makes it challenging to identify the factors influencing localization accuracy clearly. Furthermore, there is a lack of research using real equipment as a source of partial discharge. This paper presents a meta-analysis of data from monitoring systems used for radiometric localization of partial discharge sources, employing algorithms based on the time difference in arrival and received signal strength indicator. The obtained results show that the data exhibited errors with a high standard deviation, indicating that many studies are conducted under significantly different conditions. The first method is more sensitive to noise and the sampling rate, while the second is influenced by the source type and the number of sensors. Moreover, laboratory experiments performed with a potential transformer exhibiting partial discharge suggest that signal propagation in real equipment might not be omnidirectional, which can affect accuracy. Consequently, results based on controlled sources, where source directionality and obstacles have a significant impact on performance, do not accurately reflect real-world conditions. The obtained results indicate that the more realistic the source, the higher the errors will be, due to the lower uniformity in signal propagation.

## 1. Introduction

The monitoring of partial discharge (PD) activity has become a powerful predictive maintenance tool in electrical substations [1]. Research involving the study of PD is focused on four main areas. The first one addresses the electronic instrumentation involved in the detection process of the phenomenon, which includes the application of sensors such as acoustic sensors [2,3], UHF band antennas [4], and High-Frequency Current Transformers (HFCTs) [5], among others.

The second line of research is still in its early stages in the literature. It specifically focuses on the correlation between the intensity of PD signals measured by non-conventional methods and the apparent charge level of the PD [6]. However, studies have only shown the correlation linearity for certain electrode configurations [7], and it is not yet possible to generalize the results.

A third line of research focuses on classifying these signals, considering that PD can have external origins, such as surface PD and corona discharges [5], which, in general, are less critical to the integrity of insulation systems. Recent studies have addressed this through the application of machine learning techniques, such as hybrid transformer-based networks and random forest [8,9].

Finally, another major line of research, restricted to acoustic and radiometric sensors, focuses on the localization of PD sources, whether they are internal to high-voltage electrical equipment [10], such as the origin coordinates of a PD signal in a power transformer [11] or the localization of faulty equipment within an electrical substation [12].

Some of this research is dedicated to the development and evaluation of localization algorithms, which may be based on the received signal strength indicator (RSSI) [13] or on time-related information, such as the time difference of arrival (TDOA) [14] or, more recently, the Time Reversal (TR) method [15]. Among these techniques, RSSI and TDOA are the most established and widely reported in the literature, as they have been more extensively validated experimentally. In contrast, although TR presents some advantages, it generally relies on complex numerical simulations, requires phase-preserving broadband measurements, and has limited experimental validation, making it less representative for statistical analyses.

In this line of research, some studies are focused on improving instrumentation systems [1] used in the detection of radiometric PD signals, considering that localization involves the application of multiple sensors [13] and requires equipment with high bandwidth and high sampling rates [14]. Although such specifications are available in laboratory instruments, their integration into dedicated monitoring systems remains difficult and expensive.

A thorough literature analysis has shown that errors in estimating the PD source location are influenced by several factors, including the Signal-to-Noise Ratio (SNR) of the environment [14], sampling rate (SR) [16], and the instrumentation system used to acquire the signals [17], among others.

Another point worth mentioning is that tests with real equipment, especially when using algorithms based on the RSSI method, are still underexplored. In general, localization tests are conducted with controlled omnidirectional sources [18,19], which limit the effects of radiometric signal propagation, making these data less representative of commercial applications.

Therefore, the main contributions of this paper are summarized as follows: (i) a statistical analysis of monitoring data based on a systematic literature review; (ii) experimental validation using real electrical equipment as a PD source, addressing a gap in the state of the art; (iii) insights to improve the reliability of test methodologies under practical conditions. The remainder of this paper is organized as follows. Section 2 provides a brief overview of the methodology and the results of the statistical analysis conducted. Section 3 presents the results of experiments performed with a potential transformer (PT) and an omnidirectional source, along with a brief discussion. Finally, Section 4 presents the main conclusions and future works.

## 2. Statistical Analysis

The statistical research presented in this paper was conducted through a systematic review of the technical literature related to the localization of PD sources. The following aspects were observed: the localization method (RSSI or TDOA), the number of sensors, the operational range of the sensors, the type of source used to emulate the PD signal, the location of the experiment, the characteristics of the instrumentation systems, and the percentage errors. Data were collected from works published in scientific databases, such as university repositories, and recognized databases like ResearchGate, IEEE Xplore, ScienceDirect, and Google Scholar.

After analyzing the studies, statistical tools were applied to describe the data. First, the mean was calculated to determine the central tendency of the results, providing an overall understanding of the analyzed values. Next, the standard deviation was calculated to assess the variability of the data, indicating how dispersed the values were about the mean, which is crucial to defining the consistency of the presented data.

Subsequently, a data selection phase was performed using the Recursive Feature Elimination (RFE) method. This method works recursively, in which a model is adjusted, and the impact of each input on the target variable is evaluated. Therefore, the goal here was to suppress information with minimal impact on the error [20].

Next, box plots were used to visualize the data distribution clearly and intuitively. Box plots enable the quick identification of quartiles, the median, and potential outliers, facilitating the analysis of data characteristics. This graphical representation allows for the comparison of different datasets, as it provides an overall view of the variation and symmetry of the distributions.

To improve clarity and reproducibility, the following subsections present in detail the inclusion criteria applied in the selection of studies and the statistical parameters used in the comparative analysis.

### 2.1. Paper Selection

Utilization of radiometric systems for locating PD sources in electrical substations can be found as early as 1988 [21], involving measurements with two UHF sensors in a 420 kV gas-insulated substation with a 40-m length, using a single-channel digitizer, where the time delay between the trigger and captured signal determined the distance between the defect and the sensors.

In 2005 [22], a set of four antennas was utilized to locate PD sources in a 132 kV substation with dimensions of 20 × 25 m^2^. This system had a detection range limit of 15 m between the antenna and the defect origin, though it allowed for the identification of regions where more distant equipment was located.

In 2009, [12] employed a monitoring system similar to the one in Ref. [22] in high-voltage substations in the UK and the USA, detecting PD activity in PT before an explosion occurred. Comparatively, these three studies utilized UHF sensors and applied TDOA-based algorithms; however, they did not provide detailed quantitative localization results, indicating that the methodologies still require refinement. In later years, several studies applied the TDOA within smaller spatial environments. In these cases, the objective was to determine the origin (in a three-dimensional coordinate system) of the defect within the equipment itself. These studies used high-sampling-rate oscilloscopes [11], controlled point-to-plane electrode sources [14,23], and metallic environments [24] to simulate test conditions akin to those in a power transformer.

Recently, research on PD localization has increasingly focused on the electronic instrumentation involved in the detection process, with two-dimensional localization performed using algorithms based on RSSI in larger environments. The authors of [17,25] assessed envelope detection as an alternative for acquiring PD signals through simpler A/D converters, though this approach resulted in a significant reduction in information.

In the localization process, ref. [17] utilized a commercial sensing board and recommended the use of an amplifier to enhance detection resolution, taking into account the voltage drop across the diode used in envelope detection. This setup evolved over the years [1], but these two components remained present in most of the studies analyzed.

### 2.2. Data Categorization and Scope Definition

Following the state-of-the-art analysis, it was observed that a significant portion of the studies did not present quantitative data localization or failed to provide necessary information for categorization, and therefore, were not considered in the analysis [12,22].

Furthermore, studies that conducted analyses exclusively in simulation, such as [4], were also excluded, given that the errors are significantly smaller than those observed in experiments, and the quantity of such studies was insufficient for a consistent analysis. For the remaining studies, statistical tools were applied to describe and interpret the results.

The analysis is divided into two groups: the first comprises the studies that applied the TDOA method [10,11,14,23,24,26,27,28] to locate PD sources, and the second comprises the studies that applied algorithms based on RSS/RSSI methods [13,17,19,29,30,31,32,33,34].

For both groups, the output variable was the error, and the statistical analysis aimed to identify patterns and trends between the input variables and the output variable. The input variables common to both groups were the dimensional space (DS); the environment in which the experiment was conducted; the number of sensors; the sampling rate; the type of source; and the frequency range of the sensor’s detection. Specifically, for the first group, the method used to determine the time of arrival (ToA) was emphasized, and for the second group, the instrumentation used was emphasized. In summary, whether the signal was acquired from a conditioning circuit or directly from the sensor terminals.

For better data organization and a more effective comparative analysis of the results, the input variables are described in Table 1, and the data from the analyzed studies are presented in Table 2 and Table 3. The ‘quantity data’ column represents the number of location results for each author.

Following the data organization, the RFE selection technique was applied. This process enabled the selection of the most relevant variables, focusing on three variables with the most significant impact on the error, which resulted in the values presented in Table 4.

The variables with the most significant impact on the location error for the TDOA method were the environment, the method used to determine the ToA, and the sampling rate. These factors make it difficult to determine the ToA, causing location errors. The number of sensors and the detection frequency range were irrelevant factors in this analysis, since all studies used four UHF sensors and the most common frequency range for PD in power transformers [4].

Furthermore, the dimensional space did not have a significant impact on the error, as it was similar across all cases, i.e., three-dimensional and of the same order. For the RSSI method, it can be noted that the variables with the greatest impact on the location error were the signal type, the source type, and the number of sensors. The dimensional space, in this case, did not impact the error linearly due to the presence of spaces of different sizes. This analysis will be detailed in the next section.

### 2.3. Statistical Results for TDOA Method

**Figure 1:**
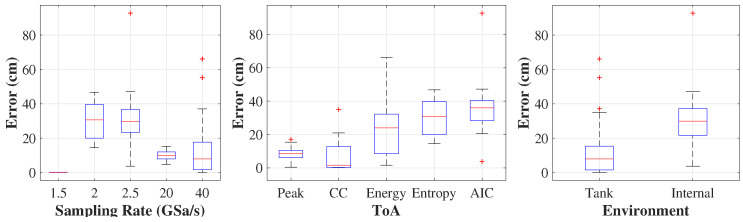
Results for TDOA.

The preliminary analysis of the errors obtained by the TDOA method indicated a mean of 20.54 cm, with a standard deviation of 16.60 cm. This deviation indicates considerable variation in the results, which is expected, given that the tests are conducted under different conditions, including variations in SNR, sampling rate, dimensional space, and method. The errors for these four variables are presented in Figure 1.

It can be observed in Figure 1 that the environment has a significant impact on the results, as a low SNR affects the determination of the arrival time and, consequently, the location [21,32]. In an environment such as a transformer tank, multipath effects are more pronounced due to the transformer core; however, this phenomenon is more relevant for the RSSI method, as it changes the signal composition and, consequently, the amplitude [4].

The sampling rate, on the other hand, was lower in cases where the error increased, making it a variable with a high impact on the TDOA method, except for [27], which is an outlier. Another variable that had a significant impact on the error was the method used to determine the ToA, where it was observed that the Cross-Correlation (CC) algorithm resulted in the smallest location errors.

### 2.4. Statistical Results for RSSI Method

**Figure 2:**
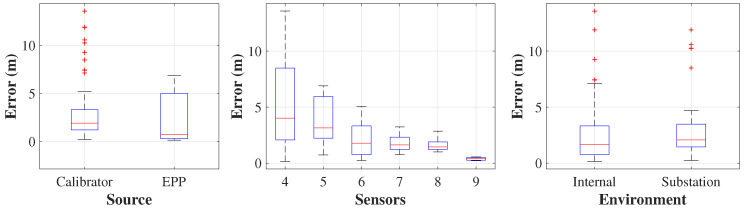
Results for the RSSI method.

The analysis of the results for the RSSI method, summarized in Table 3, revealed a mean error of 2.84 m, with a standard deviation of 2.84 m, indicating that the error varies significantly across each study, as the conditions under which each experiment was conducted differ. Figure 2 presents the box plot that shows the variation in the error associated with the selected variables.

As shown in Figure 2, the errors are higher in indoor environments. Although there is equipment in the substation that contributes to multipath effects, no obstacles were observed between the sensors, which reduces the impact on the error. It is also noted that as the number of sensors increases, both the error and variability decrease, improving the accuracy of the estimation.

Therefore, for the RSSI method, the more sensors there are, the higher the accuracy. Finally, it can be observed that the variable with the greatest influence on the error is the source type, with the highest errors associated with the EPP type source. This suggests that the more realistic the source, the higher the errors will be, due to the lower uniformity in signal propagation.

**Figure 3:**
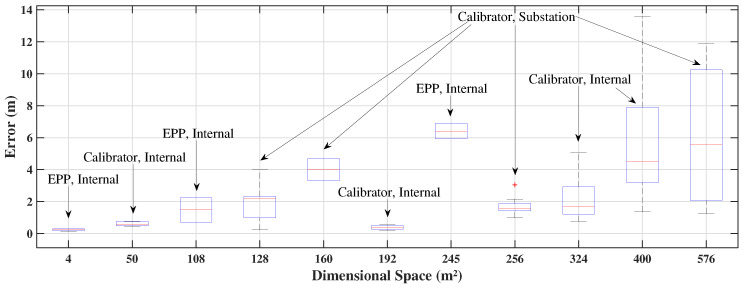
Summary of localization errors associated with the dimensional space for the RSSI method, based on the reviewed studies.

It is noteworthy that the dimensional space did not cause a linear impact on the error, as different types of sources were used and the tests were conducted in different environments, as shown in Figure 3.

However, for the same type of environment, signal, and source, the standard deviation is reduced to 1.14 m, and a higher linearity in the error is observed with the change in dimensional space.

## 3. Experimental Results

Regarding the experimental setup, two tests and a computational simulation were conducted to observe the propagation of the PD signal originating from real equipment and an omnidirectional source, as reported in some studies, and to evaluate whether the behavior is consistent for both cases.

**Figure 4:**
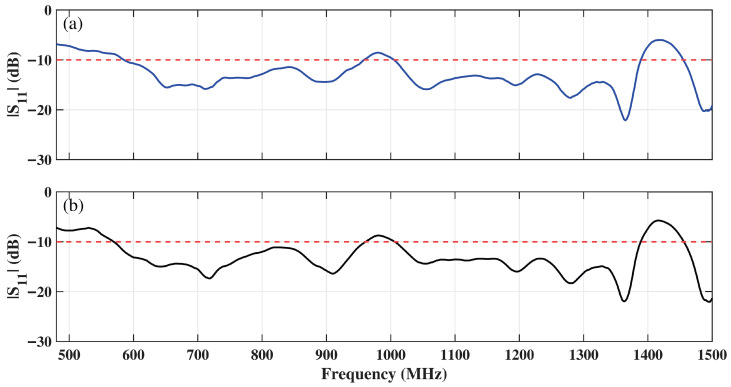
Reflection coefficients ($S_{11}$) of the two antennas used in the tests. (**a**) Antenna 1. (**b**) Antenna 2. The red dashed line indicates the −10 dB reference level.

**Figure 5:**
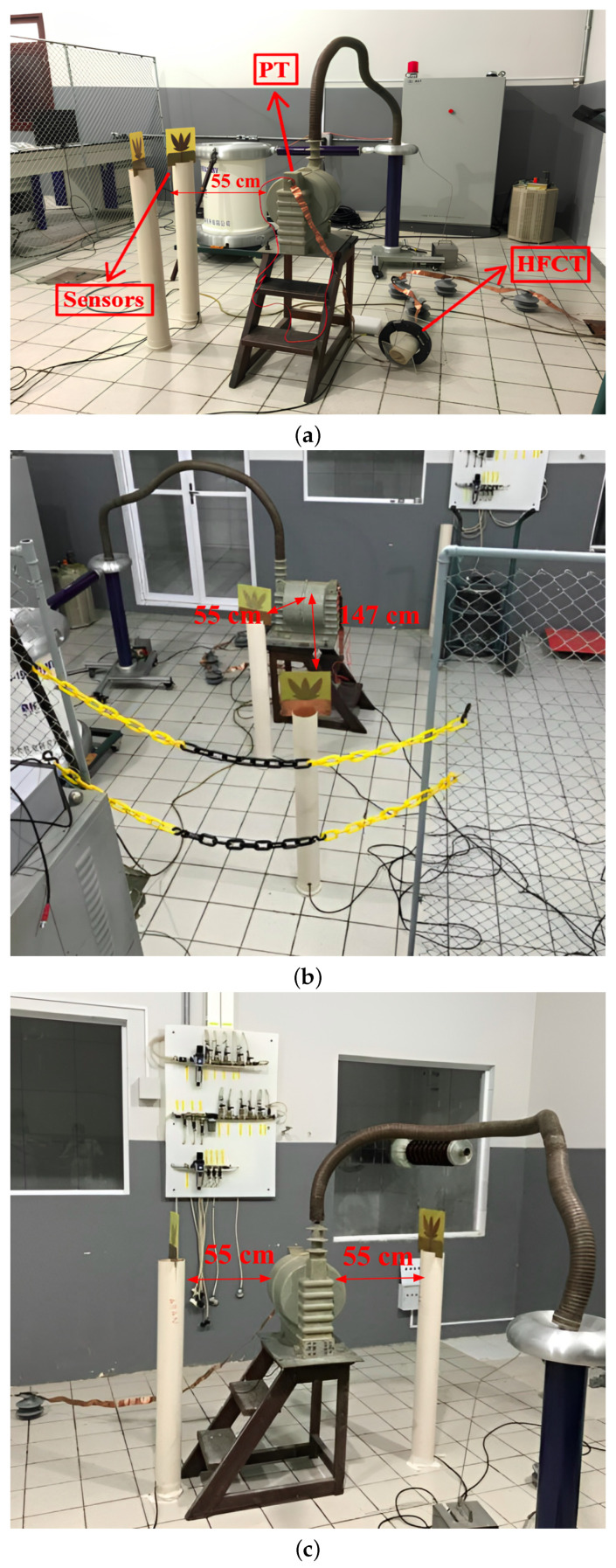
(**a**) Antennas positioned at the same distance and on the same face of the PT. (**b**) Antennas positioned at different distances but on the same face of the PT. (**c**) Antennas positioned at the same distance but on different faces of the PT.

In the setup for the first test, two nominally identical UHF microstrip patch antennas were used [4], covering the 487–1500 MHz frequency range. Each antenna consists of a leaf-shaped radiating element on an FR-4 substrate with a copper ground plane, providing an average gain of approximately 3.89 dB and an omnidirectional radiation pattern in the horizontal plane. Their reflection coefficients (S_11_) are shown in Figure 4, and the laboratory setup, which highlights the antenna placement and the 36 kV epoxy PT, is presented in Figure 5.

Signal acquisition was performed using a DSOX3014T InfiniiVision oscilloscope (Keysight Technologies, Santa Rosa, CA, USA), equipped with a 1 GHz bandwidth upgrade and operating at a sampling rate of up to 5 GS/s. The instrumentation system for PD detection included two passive High-Pass Filters (HPFs) at the antenna outputs, with a cutoff frequency of 100 MHz to suppress low-frequency noise, followed by two amplifiers and two envelope detectors. The detectors operate with RF inputs of up to 6 GHz and provide an output envelope bandwidth of up to 130 MHz [1].

In addition, a 36 kV epoxy PT (model ZI-MS94, Zilmer, São Paulo, SP, Brazil), which has a known history of insulation-related defects, was employed as a PD source [1], serving to validate the findings and demonstrate applicability to real equipment.

The antennas were positioned on different faces of the equipment, and variations in distance were made while measuring the signal intensity, as shown in Figure 5a–c. In parallel with the antenna measurements, an HFCT sensor was used to ensure that the detected signals corresponded to PD activity [17].

**Figure 6:**
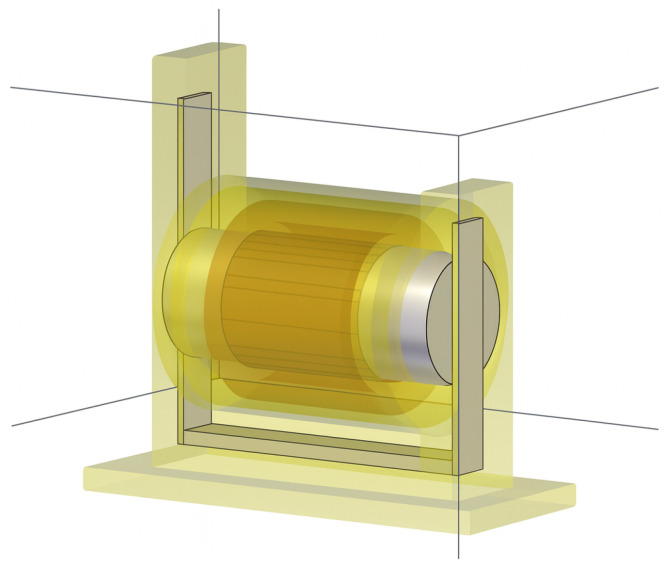
Simulated PT model.

In the computational simulation, CST Microwave Studio 2016 (Dassault Systèmes, Vélizy-Villacoublay, France) was used. This software employs the Finite Integration Technique (FIT) to solve Maxwell’s equations by discretizing them in integral form. To conduct the analysis, the PT used in the previously described test was modeled, as shown in Figure 6. Epoxy resin was used for the tank and dielectric, PEC for the core, and copper for the windings.

**Figure 7:**
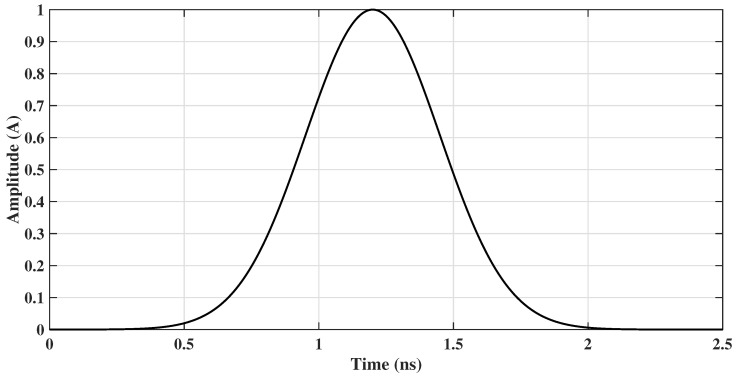
Gaussian pulse used to represent the PD signal, with a 1.5 ns width and a frequency content extending up to 1.5 GHz.

To represent the signal, a Gaussian pulse with a pulse width of 1.5 ns, a current of 1 A, and an irradiated UHF frequency range from 0 to 1.5 GHz was applied [4]. The waveform of this excitation signal is shown in Figure 7, which illustrates the temporal profile of the Gaussian pulse used to simulate the PD emission. To evaluate the UHF signal propagation within the equipment, the electric field was measured at eight different points.

**Figure 8:**
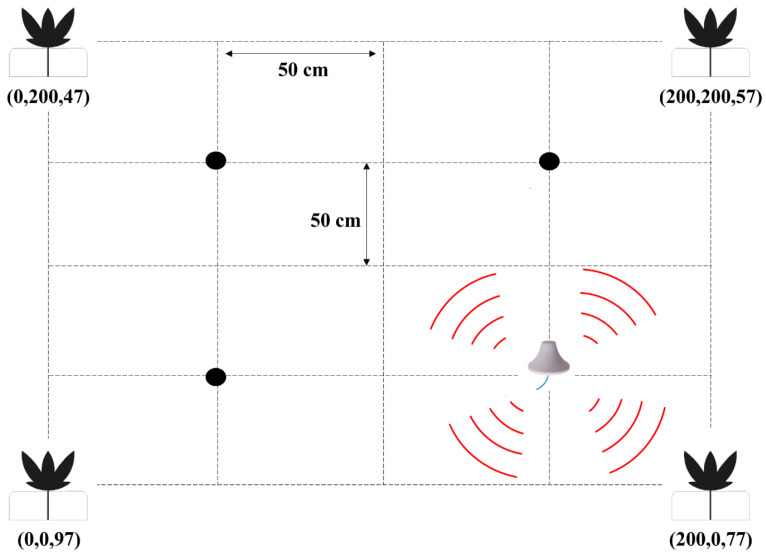
Arrangement for tests using omnidirectional source.

Finally, an experimental setup was conducted in an unobstructed environment using a commercial omnidirectional HUBER+SUHNER 1399.17.0128 antenna (HUBER+SUHNER AG, Herisau, Switzerland), connected to an Agilent N5181A signal generator (Agilent Technologies, Santa Clara, CA, USA). This antenna eliminates the need for four receivers operating in perfect synchronization during the measurement, as it radiates the same intensity omnidirectionally. The antenna was positioned at a fixed location within the setup, and the receiving antenna was moved along the four edges of the experimental setup, forming a square, as shown in Figure 8. This process was performed four times.

### 3.1. Results

**Figure 9:**
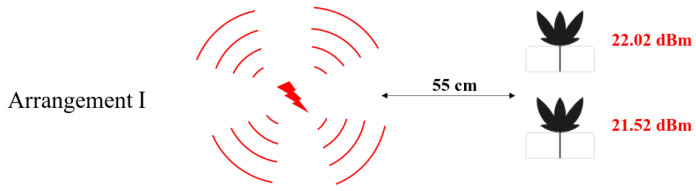
Equidistant antennas on the same face of the PT.

**Figure 10:**
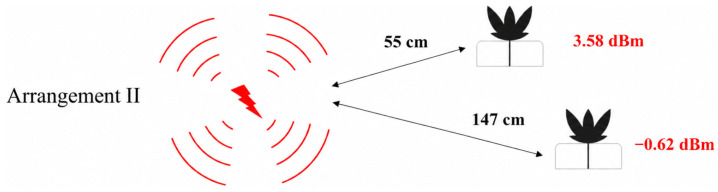
Antennas at different distances on the same face of the PT.

**Figure 11:**
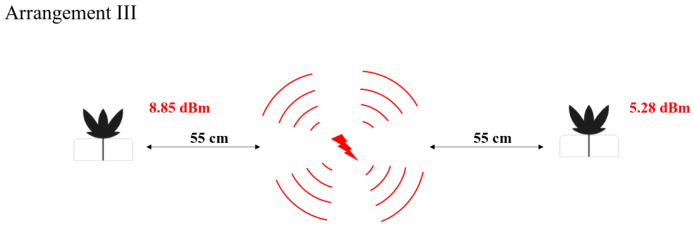
Equidistant antennas on different faces of the PT.

**Figure 12:**
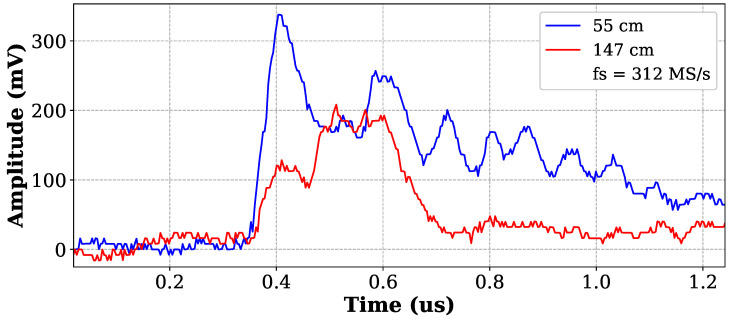
Envelope PD measurements obtained from antennas arranged at different stand-off distances on a single face of the PT.

As was previously explained, the PT was subjected to a voltage level sufficient to initiate PD activity, and two antennas were positioned at different distances and faces of the PT. The results of these measurements are presented in Figure 9, Figure 10, Figure 11 and Figure 12.

As shown in Figure 9, the antennas positioned at the same distance on the same face of the PT receive approximately the same power. When one of the antennas is moved further away, in Figure 10, the power received by the more distant antenna decreases. When the antennas are at the same distance from the source but on different faces of the PT, Figure 11, a variation in the received power is observed, indicating that the propagation is not omnidirectional, as there are reflections and refractions inside the PT. This suggests that location estimation carried out using the RSSI method is more prone to errors.

Additionally, as shown in Figure 12, the envelopes do not provide a precise determination of the wavefronts. Consequently, the principle of Fermat, which assumes that the propagation path corresponds to the minimum time of travel, cannot be directly applied to these signals. As summarized in Table 2, most RSSI-based studies adopt envelope detection instead of wavefront detection, which limits the applicability of time-of-arrival approaches.

**Figure 13:**
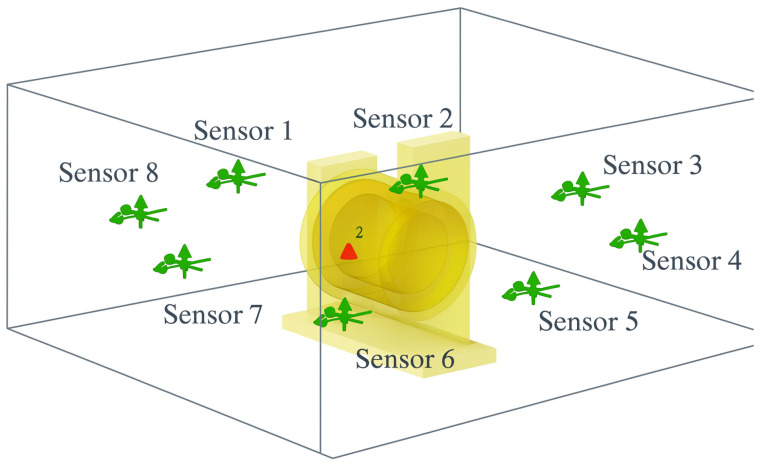
Spatial arrangement of the sensors in PT, simulated with CST Microwave Studio software.

**Figure 14:**
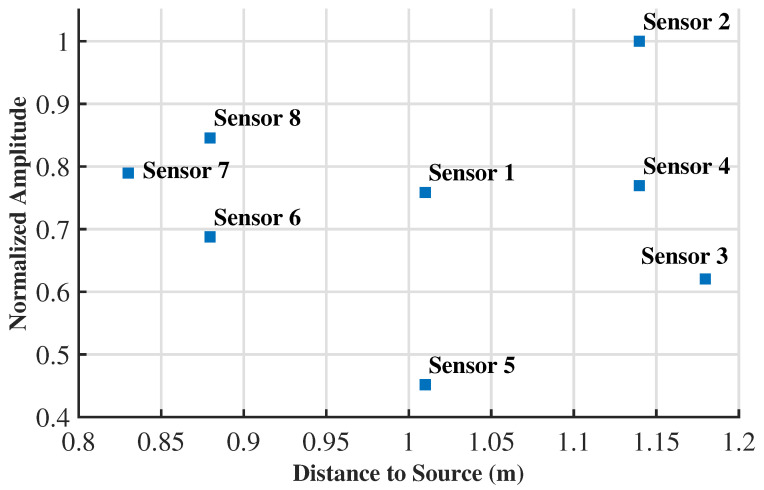
Sensor intensity received in computational simulation using CST Microwave Studio software.

Figure 13 presents the PT and the spatial arrangement of the sensors used in the simulation, and Figure 14 shows the signal intensity received by each of the eight sensors.

**Figure 15:**
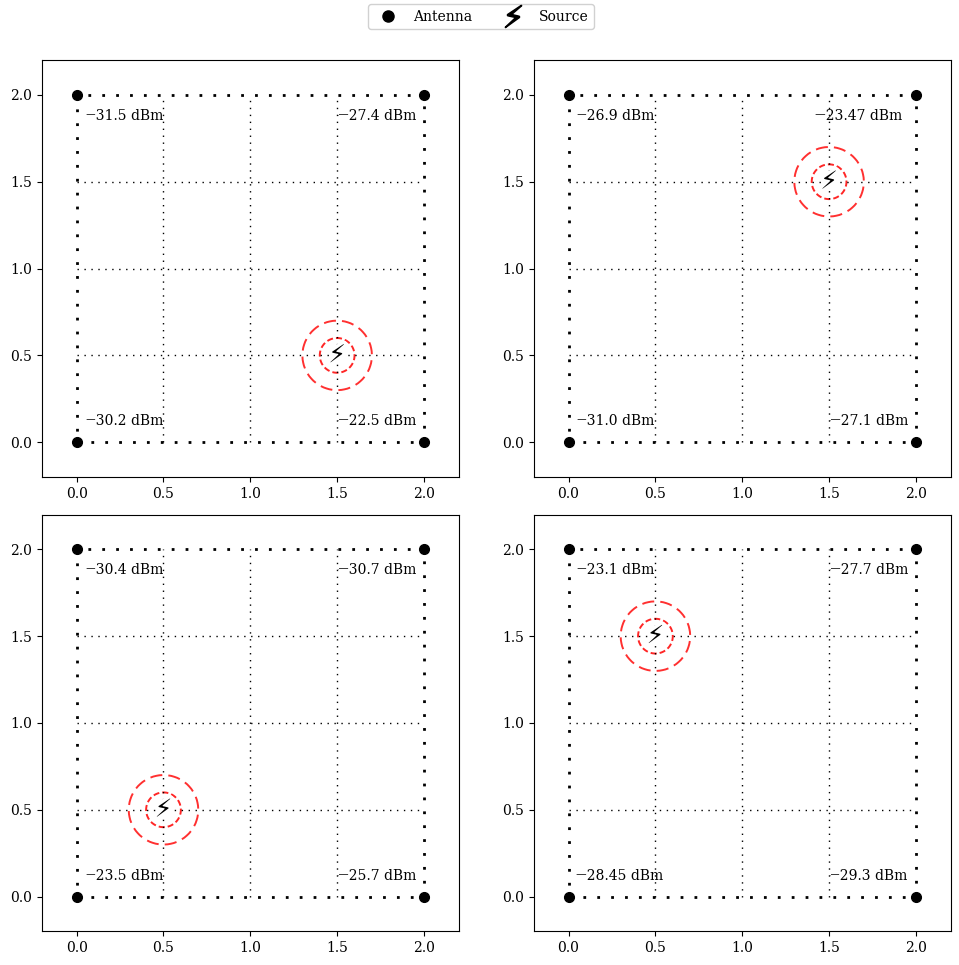
Signal intensity received for the arrangement with omnidirectional source at four locations.

As observed in the experiment, the RSSI of each sensor does not only depend on the distance from the sensor to the source, indicating that there is no omnidirectional propagation. This occurs due to multiple internal reflections, diffractions, and attenuations within the PT, which modify the trajectory and intensity of the pulses, underscoring the need for a substantial number of sensors to overcome these issues. The results of the tests using omnidirectional source are presented in Figure 15.

As shown in Figure 15, the signal intensity is observed to depend solely on the distance from the sensor to the source, which differs from the behavior observed for the transformer. This experiment is important for evaluating the algorithms; however, it does not represent what is typically observed in real equipment.

### 3.2. Discussion

RSSI-based methods are often adopted as an alternative to TDOA, which can achieve high accuracy but require high sampling rates, as indicated by the meta-analysis. However, a critical point of discussion addresses why the RSSI method is more susceptible to error than the TDOA method when the emitting source is not omnidirectional. The TDOA method relies on Fermat’s principle, which governs the path of least propagation time between the source and the sensors. Consequently, localization errors due to source directivity or obstacles tend to be smaller, since time delays are less sensitive to these variations, as demonstrated by [4].

In contrast, methods based on RSSI depend on measuring the signal’s attenuation during propagation. This attenuation is significantly influenced by factors such as source directivity (or radiation pattern), reflections, and medium dispersion, rendering the RSSI method more prone to errors than the TDOA method. The impact of signal attenuation on distance estimation is substantially greater than that of variation in time of arrival on TDOA.

Experimental measurements, supported by simulations, showed that the PT used in this study does not exhibit isotropic radiation, representing only one of the many geometries found in real equipment. It is essential to note that many studies in the literature have validated the RSSI method using omnidirectional sources. Consequently, these methodologies, where source directivity and obstacles significantly impact the method’s performance, may not accurately reflect real-world conditions. These effects should be taken into account in localization tasks, and future studies should determine whether sensor redundancy or angularly distributed antenna arrangements can help mitigate the errors introduced by non-omnidirectional emission.

## 4. Conclusions

A statistical analysis of the results from research on radiometric localization of PD was presented. Significant deviations were observed in the results, indicating that the experiments were conducted under different conditions, which caused a substantial impact on the error. For the TDOA method, the most relevant variables were the sampling rate, the environment’s SNR, and the method used to determine the ToA. For the RSSI method, the variables that most affected the error were the source type, the number of sensors, and the dimensional space.

Furthermore, although studies using the RSSI method have made significant advancements in algorithm development and employed instrumentation, there is still a need for experiments that better represent the propagation in real equipment. This is because experimental tests have shown that signal propagation inside equipment is not omnidirectional, contrary to what has been reported in most studies. Future experiments using different equipment and additional sensors will be conducted to refine the localization methodology.

## Figures and Tables

**Table 1:** Variable categorization.

Input Variable	Label	Description
Environment	Internal	Laboratory and similar facilities
Environment	Tank	Power transformer tank
Source	EPP	Point-plane and needle-ring electrodes immersed in oil or air
Source	Calibrator	Calibrators and similar PD emitters

**Table 2:** Overview of TDOA-related studies and their main characteristics.

Author	Source	Environment	Sensors	DS (m^3^)	SR (MS/s)	Frequency Band (MHz)	ToA Method	Quantity of Data
[11]	EPP	Tank	4	0.80	40,000	300–1500	Peak, Energy, Entropy, CC, AIC	36
[23]	EPP	Internal	4	2.00	2000	300–1500	Entropy	10
[14]	EPP	Internal	4	3.88	2500	487–1500	Energy	32
[10]	EPP	Internal	4	3.88	2500	487–1500	Peak, Energy	8
[27]	EPP	Tank	4	9.73	1500	300–1500	CC	1
[26]	EPP	Tank	4	24.34	20,000	340–440	Energy, CC	6
[35]	EPP	Internal	4	3.88	2500	487–1500	AIC	4
[28]	EPP	Tank	4	0.02	20,000	300–1500	Energy	15
[27]	EPP	Tank	4	19.00	1500	300–1500	CC	4

**Table 3:** Overview of RSS/RSSI-related studies and their main characteristics.

Author	Source	Environment	Sensors	DS (m^2^)	Signal	SR (MS/s)	Frequency Band (MHz)	Quantity of Data
[13]	Calibrator	Internal	6	324	Conditioning	1.1	30–320	9
[36]	Calibrator	Internal	8	324	Conditioning	2000	30–320	2
[13]	Calibrator	Internal	7	324	Conditioning	1.1	30–320	9
[13]	Calibrator	Internal	8	324	Conditioning	1.1	30–320	9
[17]	EPP	Internal	5	245	Integrity	10,000	200–2000	2
[17]	EPP	Internal	5	108	Conditioning	10,000	200–2000	2
[19]	Calibrator	Internal	8	324	Conditioning	1000	30–320	1
[19]	Calibrator	Substation	6	324	Conditioning	1000	30–320	1
[13]	Calibrator	Substation	5	128	Conditioning	1.1	30–320	2
[13]	Calibrator	Substation	6	128	Conditioning	1.1	30–320	4
[13]	Calibrator	Substation	7	256	Conditioning	1.1	30–320	3
[13]	Calibrator	Substation	8	256	Conditioning	1.1	30–320	3
[13]	Calibrator	Substation	6	160	Conditioning	1.1	30–320	2
[13]	Calibrator	Substation	7	256	Conditioning	1.1	30–320	2
[13]	Calibrator	Substation	8	256	Conditioning	1.1	30–320	2
[33]	Calibrator	Internal	6	50	Conditioning	2000	30–320	4
[33]	Calibrator	Internal	6	50	Conditioning	2000	30–320	2
[34]	Calibrator	Substation	4	576	Conditioning	2.7	300–1500	10
[32]	EPP	Internal	4	4	Conditioning	2500	487–1500	3
[31]	Calibrator	Internal	4	400	Conditioning	2.7	300–1500	13
[30]	Calibrator	Internal	9	192	Conditioning	2.7	300–1500	4
[36]	Calibrator	Substation	8	256	Conditioning	2000	30–320	2

**Table 4:** Selected variables for statistical analysis.

Method	Variable 1	Variable 2	Variable 3
TDOA	Environment	Sampling Rate	ToA Method
RSSI	Source	Sensors	Environment

## Data Availability

The data presented in this study are available on request from the corresponding author.
